# High Throughput Correlative Electrochemistry-Microscopy Analysis on a Zn–Al Alloy

**DOI:** 10.1021/acsphyschemau.4c00016

**Published:** 2024-05-10

**Authors:** Gunani Jayamaha, Levi Tegg, Cameron L. Bentley, Minkyung Kang

**Affiliations:** † School of Chemistry, The University of Sydney, Camperdown, New South Wales 2006, Australia; ‡ School of Aerospace, Mechanical and Mechatronic Engineering, The University of Sydney, Camperdown, New South Wales 2006, Australia; § School of Chemistry, Monash University, Clayton, Victoria 3800, Australia

**Keywords:** scanning electrochemical cell microscopy, correlative analysis, heterogeneous electrochemical reaction, electrochemical metal dissolution, metal alloys, corrosion

## Abstract

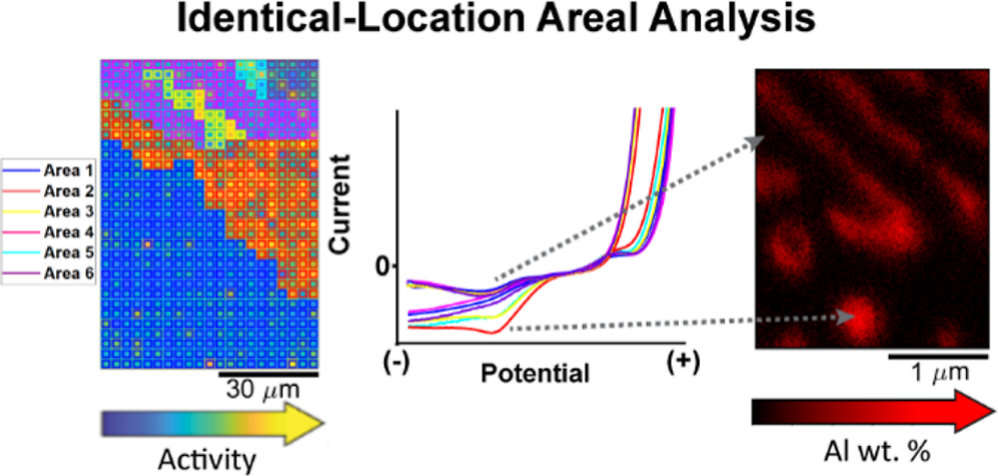

Conventional electrodes and electrocatalysts possess complex compositional and structural motifs that impact their overall electrochemical activity. These motifs range from defects and crystal orientation on the electrode surface to layers and composites with other electrode components, such as binders. Therefore, it is vital to identify how these individual motifs alter the electrochemical activity of the electrode. Scanning electrochemical cell microscopy (SECCM) is a powerful tool that has been developed for investigating the electrochemical properties of complex structures. An example of a complex electrode surface is Zn–Al alloys, which are utilized in various sectors ranging from cathodic protection of steel to battery electrodes. Herein, voltammetric SECCM and correlative microstructure analysis are deployed to probe the electrochemical activities of a range of microstructural features, with 651 independent voltammetric measurements made in six distinctive areas on the surface of a Zn–Al alloy. Energy-dispersive X-ray spectroscopy (EDS) mapping reveals that specific phases of the alloy structure, particularly the α-phase Zn–Al, favor the early stages of metal dissolution (i.e., oxidation) and electrochemical reduction processes such as the oxygen reduction reaction (ORR) and redeposition of dissolved metal ions. A correlative analysis performed by comparing high-resolution quantitative elemental composition (i.e., EDS) with the corresponding spatially resolved cyclic voltammograms (i.e., SECCM) shows that the nanospot α-phase of the Zn–Al alloy contains high Al content (30–50%), which may facilitate local Al dissolution as the local pH increases during the ORR in unbuffered aqueous media. Overall, SECCM-based high-throughput electrochemical screening, combined with microstructure analysis, conclusively demonstrates that structure-composition heterogeneity significantly influences the local electrochemical activity on complex electrode surfaces. These insights are invaluable for the rational design of advanced electromaterials.

## Introduction

Zinc–aluminum (Zn–Al) alloys are versatile materials that offer a combination of mechanical strength, high tunability in their composition and microstructure, and unique electrochemical properties, making them suitable for a wide range of applications, ranging from corrosion-resistant coatings for structural steel to electromaterials for Zn-based metal–air batteries.^1,2^ Zn–Al alloys provide superior anodic corrosion protection for steel,^3,4^ as they lead to the formation of corrosion products that are less soluble and more protective.^5^ For application in Zn–air batteries, alloying Al with Zn provides a higher open circuit potential compared to pure Zn,^6,7^ leading to an increase in the specific capacity of the electrode and offering benefits in terms of anodic specific capacity and reduced electrode weight.

As-cast Zn–Al alloys exhibit a coarse and heterogeneous microstructure with large grains and inclusions possessing various compositional profiles due to the rapid solidification process that occurs during casting.^8,9^ In contrast, annealed Zn–Al alloys display a more homogeneous microstructure as a result of the heat treatment process that is being employed.^10,11^ In practical applications in metal coating or battery electrodes, as-cast Zn–Al alloys are often used, so there is significant interest in correlating the electrochemical performance of the alloy with the microstructural surface features and/or composition.^12,13^

A number of studies have been carried out to explore the influence of both macro- and microstructures on the mechanical properties of both hyper-eutectic and hypo-eutectic Zn–Al alloys in their as-cast state.^14^ These studies mostly centered around their impact on mechanical properties, including hardness, elongation, and yield stress.^13,15^ While prior research has extensively explored the mechanical characteristics of Zn–Al alloys, there have been relatively few studies that specifically investigated their electrochemical properties. The few that are available primarily focused on the bulk properties of the alloys, employing conventional electrochemical analyses. However, the emphasis remained on the bulk characteristics,^16−18^ and little attention was given to evaluating the local microstructure.^5,19^ Given the extensive application of Zn–Al alloys, particularly due to their advantageous mechanical and electrochemical properties, there is a critical need for studies that provide a more comprehensive local-level electrochemical analysis. Such detailed examinations can greatly enhance our understanding of the underlying mechanisms governing the overall (bulk) electrochemical properties.

Conventional macroscopic electrochemical characterization performed on the bulk material provides limited information about structure–activity relationships in complex materials such as Zn–Al alloy due to the disparate scales of measurement (i.e., mm) and microstructure (i.e., nm to μm). Scanning electrochemical cell microscopy (SECCM) has emerged as a premiere electrochemical imaging method that enables local structure–activity analysis on electrode surfaces.^20−23^ The SECCM probe provides a mobile electrochemical cell with dimensions of nanometer resolution, allowing electrochemical measurement to be directly carried out at the microstructure of the interest.^24,25^ Combined with multiple microscopic techniques carried out in an identical-location manner, SECCM is able to directly resolve the relationship between electrode structure and electrochemical function (e.g., activity).^26,27^ Localized electrochemical analysis utilizing SECCM has been performed on metal alloys including annealed Zn–Al alloys.^19,28,29^ However, as-cast metal alloys possessing more complex surface structures have not yet been explored.

This study aims to provide insights into the local electrochemical properties of a hypoeutectic Zn-4 wt. %Al alloy in its as-cast state when exposed to unbuffered, neutral pH media. The use of SECCM as the primary investigative tool allows for comprehensive local electrochemical analysis of the alloy. Complementary microscopy techniques, including scanning electron microscopy (SEM) and energy-dispersive X-ray spectroscopy (EDS), are utilized to perform correlative structural characterizations of the surface microstructure and composition. This approach seeks to offer a deeper understanding of the electrochemical behavior of Zn–Al alloy within the context of its unique microstructure and composition.

## Experimental Section

### Chemicals

Sodium chloride (NaCl, anhydrous, ≥99.0%, Sigma-Aldrich), potassium chloride (KCl, 99.5%, Sigma-Aldrich), dodecane [CH_3_(CH_2_)_10_CH_3_ ≥ 99.0%, Sigma-Aldrich], and Ag wire (99.99%, 0.125 mm in diameter, Goodfellow Cambridge Ltd., UK) were used as supplied by the manufacturer. All aqueous solutions were prepared with deionized water (Milli-Q Reference Water Purification System, 18.2 MΩ cm resistivity at 25 °C). Dodecane was employed to create an oil layer on the metal sample during SECCM scanning (vide infra).

### Probe and Substrate Preparation

To prepare the SECCM probes, borosilicate glass capillaries with a filament (1B120F-4, outer diameter of 1.2 mm and inner diameter of 0.68 mm, World Precision Instruments, USA) were pulled using a two-stage capillary gravity-puller (PC-100, Narishige, Japan). The setup parameters for a probe tip diameter of 400 nm were heat of 56 and weight of 4 in a single step. The pipet orifice was characterized using SEM, revealing an approximate diameter of 400 nm (Figure S1 in the Supporting Information) with high reproducibility. Each prepared pipet was filled with a 10 mM NaCl solution, leaving a small volume of silicone oil at the top to prevent electrolyte evaporation during the typically hour-long scan. The quasi-reference counter electrode (QRCE) for the SECCM probe was prepared by anodizing Ag wire (Goodfellow Cambridge Ltd., UK) in a saturated KCl solution, resulting in AgCl-coated Ag. This QRCE was then inserted into the pipet filled with the electrolyte. The AgCl-coated Ag wire was calibrated against a commercial leak-free Ag/AgCl electrode (eDAQ, Australia; 3.4 M KCl) through open-circuit potential measurements, and all potentials are presented relative to the Ag/AgCl (3.4 M KCl) scale.

The overall elemental composition of the as-cast Zn–Al alloy, containing 96 wt. % Zn and 4 wt. % Al, was determined by inductively coupled plasma–mass spectrometry (PerkinElmer, NexION 350X). Approximately, a 1 × 1 × 1 cm^3^ piece of the alloy was mounted at room temperature on a carbon mixed resin (EpoFix, Struers) sample holder (Figure S2 in the Supporting Information). The sample holder was laterally drilled until it reached the alloy, establishing direct electrical contact with a copper wire (Altronics, Taiwan). The copper wire was firmly affixed with silver paint and epoxy glue, guaranteeing a robust electrical connection between the alloy and the copper wire.

To achieve a smooth and uniform substrate surface, mechanical polishing was conducted using a TegraPol-25 polishing wheel and SiC papers (Struers, Canada) with increasing grit sizes, concluding with the final step of chemical-mechanical polishing using an OP-S suspension (Struers, Canada). Following the final polishing step, the sample was sonicated in anhydrous ethanol for 2 min to remove any remaining residue. To enable subsequent identical-location surface analysis after SECCM measurements (vide infra), a mark was created by scratching the alloy surface with a surgical blade (Figure S2 in the Supporting Information).

A ring of Quick Set Epoxy (RS PRO, Australia) was positioned around the upper edge of the sample to secure the thin and consistent layer of dodecane. This minimized SECCM meniscus evaporation and ensured droplet stability during scanning.^30^ In order to characterize the surface structure and local elemental composition of the alloy, SEM and EDS were performed on a Zeiss Sigma HD FESEM using a 10 kV incident beam and an Oxford Instrument X-Max EDS detector, respectively. Images were collected using an Everhart-Thornley secondary electron detector and a quadrant electron backscatter detector (BSE). Element maps were generated from the EDS data by using the Oxford Instruments AZtec software package.

### SECCM Operation

The voltammetric SECCM was operated within a home-built SECCM setup that utilized a single-barrel nanopipette as a SECCM probe, similar to the previous reports.^31−34^ The probe was mounted on an XYZ nanopositioner (Nano-3D200, MCL Inc., USA) using a custom-made probe holder, enabling nanoscale spatial resolution for probe translation. For coarse translational adjustments of the probe over micrometer to centimeter scales, a micropositioner (M370, Biologic, France) was employed. In the experiment, a prepared Zn–Al alloy sample served as the working electrode (WE), while an AgCl-coated Ag electrode was used as the QRCE. Pipette positioning was guided by monitoring the probe position with a camera (Chameleon3, FLIR; equipped with a 2× objective lens from Edmund Optics) and by referencing the marks created on the sample surface (vide supra). This visual monitoring of the probe positioning process ensured that the meniscus was fully immersed in the dodecane layer spread over the alloy sample.

For the voltammetric SECCM experiment, the approach voltage was set to −0.8 V versus Ag/AgCl to provide electrochemical positional feedback when the meniscus made contact with the alloy surface. Once the meniscus contacted the surface, a cyclic voltammogram (CV) was acquired in the positive and negative directions, ranging from −1.33 to −0.6 V, at a scan rate of 0.5 V s^–1^. The scan area covered 60 × 90 μm^2^, with a hopping distance of 3 μm, resulting in the collection of 651 individual CVs. The data acquisition was managed through an FPGA card (NI USB-7855R from National Instruments, USA), which serves as the intermediary between the LabVIEW software program (National Instruments, USA) and the SECCM setup that is overall controlled by the Warwick electrochemical–scanning probe microscopy (www.warwick.ac.uk/electrochemistry) software platform. Subsequently, the data were processed using MATLAB (MathWorks, USA) programs customized for varied data analysis and representation. Additional data analysis was performed using an OriginPro 2023b 10.0 (OriginLab, USA).

## Results and Discussion

### Voltammetric SECCM: Operational Details and Considerations

**Figure 1:**
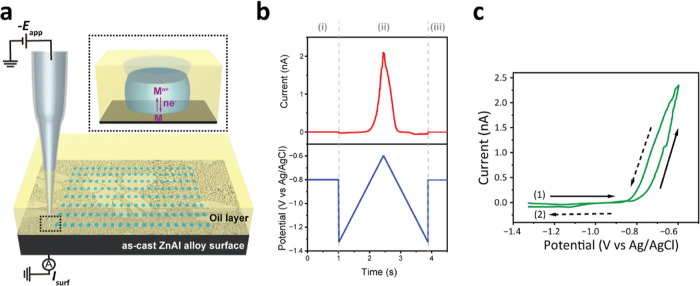
(a) Schematic representation of the voltammetric SECCM scan on the Zn–Al alloy surface, showing the dodecane oil layer that is added to ensure droplet stability during the scan and the advancing droplet meniscus that penetrates the oil layer to reach the alloy substrate. Applied potential (*E*_app_) and surface current (*I*_surf_) are indicated. (b) A graph illustrating *I*_surf_ and *E*_app_ over time at each hop: (i) the SECCM probe approaches at *E*_app_ of −0.8 V until detecting meniscus contact with the alloy surface when *I*_surf_ surpasses a threshold of 1.9 pA; (ii) cyclic voltammetry is performed between −1.33 and −0.6 V at a scan rate of 0.5 V s^–1^ following a brief rest of 1 ms after achieving meniscus contact at −1.33 V; (iii) probe is retracted from the initial position to move to the next defined location. (c) Example CV acquired during a scanning step, with (1) indicating the initial sweep in the positive direction and (2) the reverse sweep in the negative direction.

Voltammetric SECCM^35^ was conducted in the hopping scan mode, as depicted in Figure 1. This mode involves acquiring individual voltammetric (cyclic voltammetry) measurements at predetermined positions within a defined array (Figure 1a). The electrolyte meniscus from the SECCM probe makes contact with the Zn–Al alloy surface by locally displacing the oil layer, which is added to maintain droplet stability throughout the scan.^28,30,34^ To ensure that individual measured areas do not overlap (i.e., each voltammetric measurement is independent of the last), despite using a nanopipet (approximately 400 nm in diameter) as the SECCM probe, the distance between two adjacent electrochemical measurements, known as the hopping distance, was maintained at 3 μm in both the *x* and *y* directions during the scan of the Zn–Al alloy surface in the array. Herein, a total of 651 CVs were acquired across a scan area of 60 × 90 μm^2^.

Upon each landing of the SECCM probe, the meniscus cell establishes contact with the metal alloy surface at an applied voltage (*E*_app_) of −0.8 V vs Ag/AgCl. This closes the electrochemical circuit and causes a current to flow which exceeds the predefined feedback threshold of 1.9 pA, signaling the probe translation to halt [see Figure 1b(i)]. *E*_app_ is immediately switched to −1.33 V vs Ag/AgCl and remains constant for 1 ms at the surface, allowing the nonfaradaic (capacitive) current to decay. Subsequently, a CV is acquired at a scan rate of 0.5 V s^–1^, sweeping the potential to −0.6 V vs Ag/AgCl before returning to −1.33 V [see Figure 1b(ii)]. Completing one cycle of cyclic voltammetry measurement, the pipet is vertically retracted from the measurement point to be relocated to the subsequent position [see Figure 1b(iii)]. The potential window, ranging from −1.33 to −0.6 V, encompasses the oxygen reduction reaction (ORR) from −1.33 to −1.1 V,^36,37^ followed by the metal dissolution that starts at −0.8 V and persists until −0.6 V [Figure 1c(1)]. In the reverse direction, subsequently, metal redeposition and ORR occurs near −1.1 V, ultimately returning to predominantly ORR as more negative *E*_app_ is applied [Figure 1c(2)] (Figure S6 in the Supporting Information). Considering that the charge between −0.8 and −0.6 V in the positive direction (i.e., metal dissolution) is much larger than the charge at the peak near −1.1 V (i.e., metal redeposition and ORR), a majority of the dissolved metal ions are not recovered to the alloy surface.^38^ The complete recovery of the dissolved metal ions is not achievable, primarily due to the high mass transport rate^39^ within the meniscus at the nanoscale and the time scale of the individual voltammetric measurements.

### Microstructure Analysis on As-Cast Zn–Al Alloys

**Figure 2:**
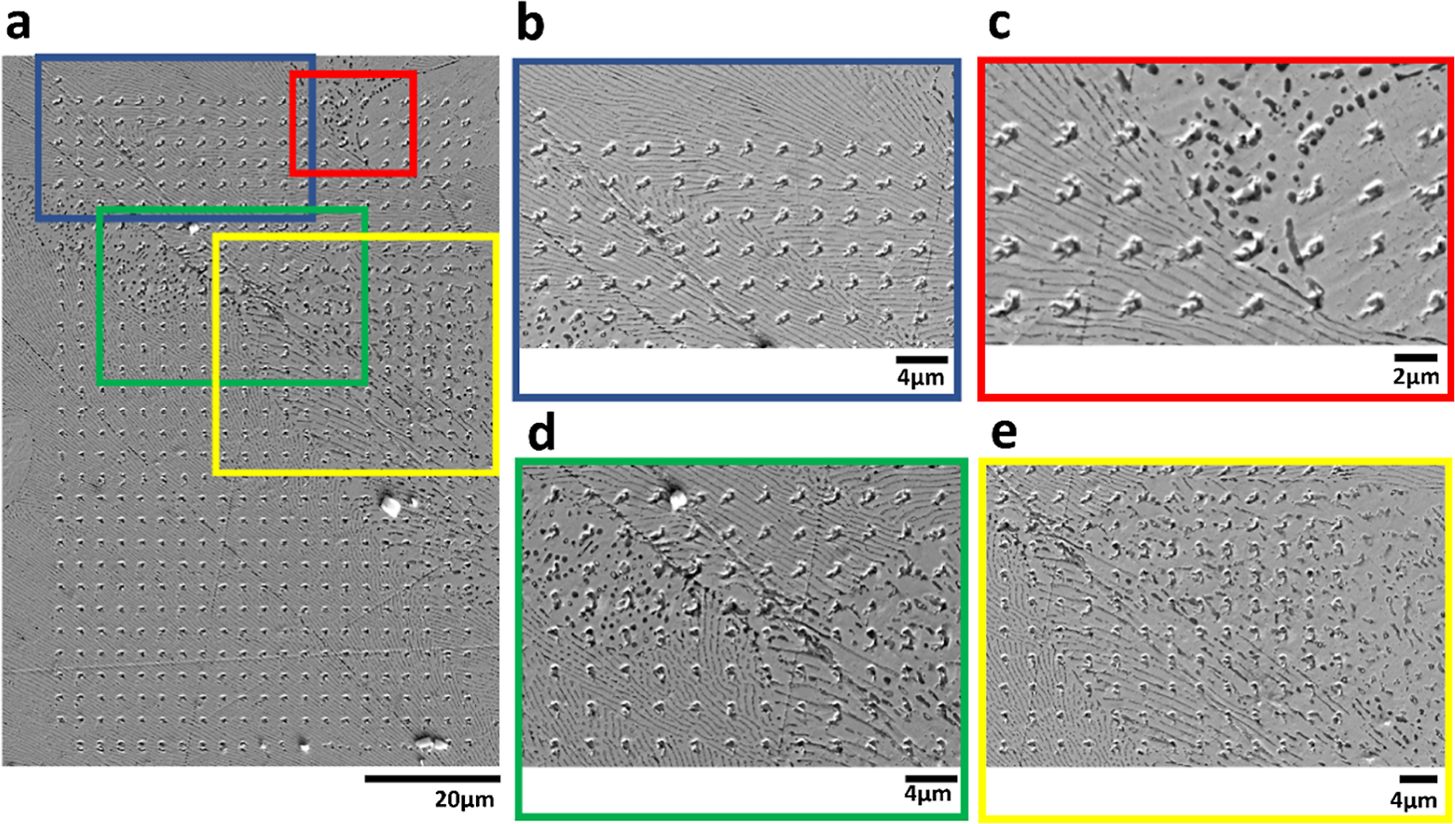
(a) SEM image in SE2 mode of the complete SECCM scan area, representing indents created by metal dissolution during each “hop” of the scan, with a total of 651-point measurements covering various microstructures on the Zn-4 wt. % Al alloy. (b) Area highlighted by the blue box reveals the lamellar structures of the Zn-rich β phase (lighter color) and the Zn–Al eutectic γ phase (darker color). (c) Light-colored island is observed in the right corner, identified as a Zn-rich β phase. Additionally, this SEM image contains dark nanospots (approximately 0.4 μm diameter) within a light background. These spots represent the Al-rich α phase embedded within the Zn-rich β phase. The overlapping lamellar structure on the left is highlighted by the red box. (d,e) Al-rich α phase nanospots in various regions of the alloy adjacent to the lamellar structure are represented by green and yellow boxes, respectively.

The voltammetric SECCM technique was utilized to conduct a comprehensive electrochemical analysis of the Zn–Al alloy, capitalizing on its main advantage of performing correlative microscopy analysis, initially with colocated SEM. The surface region of interest was identified after SECCM scanning using two SEM imaging modes: secondary electron (SE2) and backscattered electron (BSE). The SEM image in Figure 2a displays indents, with each corresponding to a metal dissolution event that took place within the meniscus cell during each “hop” of the voltammetric SECCM experiment (vide supra). A set of 20 droplet footprints in the SEM image (Figure 2c) were analyzed to determine the mean droplet footprint size, which have a mean diameter of 1.54 μm, with a standard deviation of 0.14 μm. Current density (*J*; mA cm^–2^) was calculated by normalizing the current (*I*_surf_; nA) collected from SECCM with the averaged footprint area obtained from the size analysis.

Note that the SE2 mode was utilized in Figure 2 to precisely determine the location of the SECCM scan. This mode offers high sensitivity to the surface morphology while providing moderately qualitative information regarding the elemental composition. This sensitivity allows for the identification of droplet footprints from SECCM measurements of specific microstructures within the as-cast Zn–Al alloy. In contrast, the BSE mode, as shown in Figure S4 in the Supporting Information, is more responsive to the atomic number of the elements, resulting in higher contrast and clearer qualitative information regarding variations in elemental composition compared to the SE2 mode.^40^

The microstructural characteristics observed in the hypo-eutectic alloy are primarily attributed to the presence of the Al-rich α phase (face-centered cubic, fcc), Zn-rich β phase (hexagonal close-packed), and ZnAl γ phase (fcc), in accordance with the Zn–Al phase diagram.^41^ The phase transition of Zn-4 wt. % Al as a function of temperature is explained in detail in the Supporting Information, Section S2. While dendritic Zn-rich islands enclosed by Zn and Al lamellar structures are commonly observed in hypo-eutectic Zn–Al alloys (Figure S3 in the Supporting Information), the as-cast Zn–Al alloy studied herein exhibits diverse surface microstructure.^42^

Referring back to Figure 2a, the as-cast Zn–Al alloy exhibits a complex microstructure with three primary microstructural characteristics being identified. Figure 2b represents an area with lamellar structures, showing layers of the Zn-rich β phase with average thickness of 0.28 μm (lighter color) and ZnAl eutectic γ phase with average layer thickness of 0.13 μm (darker color). Note that there is a grain boundary (GB) between the two distinctive lamellar structures. In Figure 2c, a light-colored dendritic island, typically tens of micrometers in size, is observed in the right corner. This island is identified as a Zn-rich β phase. This SEM image also includes dark nanospots with approximate diameters of 0.4 μm among a light background, comprising the Al-rich α phase embedded within the Zn-rich β phase, adjacent to the overlapping lamellar structure on the left seen in Figure 2b. Figure 2d,e depicts additional Al-rich α phase nanospots in various regions of the alloy that are adjacent to the lamellar structure.

Based on previous studies, binary hypoeutectic Zn–Al alloys typically exhibit Zn-rich β phase islands surrounded by ZnAl eutectic γ phase and Al-rich α phase combined with the Zn-rich β phase.^43^ The Zn-rich β phase has been shown to possess an elemental composition of 99% Zn and 1% Al (based on weight percentage) through EDS analysis. The darker ZnAl eutectic γ phase often contains 73–78% Zn and 23–26% of Al. The Al-rich α phase nanospots display a composition ranging from 35 to 50% of Al within the Zn-rich β phase, with 78–88% of Zn and 11–23% of Al.^44,45^ Note that detailed quantitative elemental analysis of the as-cast Zn–Al alloy used herein is discussed in a later section.

### Local Electrochemical Analysis on As-Cast Zn–Al Alloys

**Figure 3:**
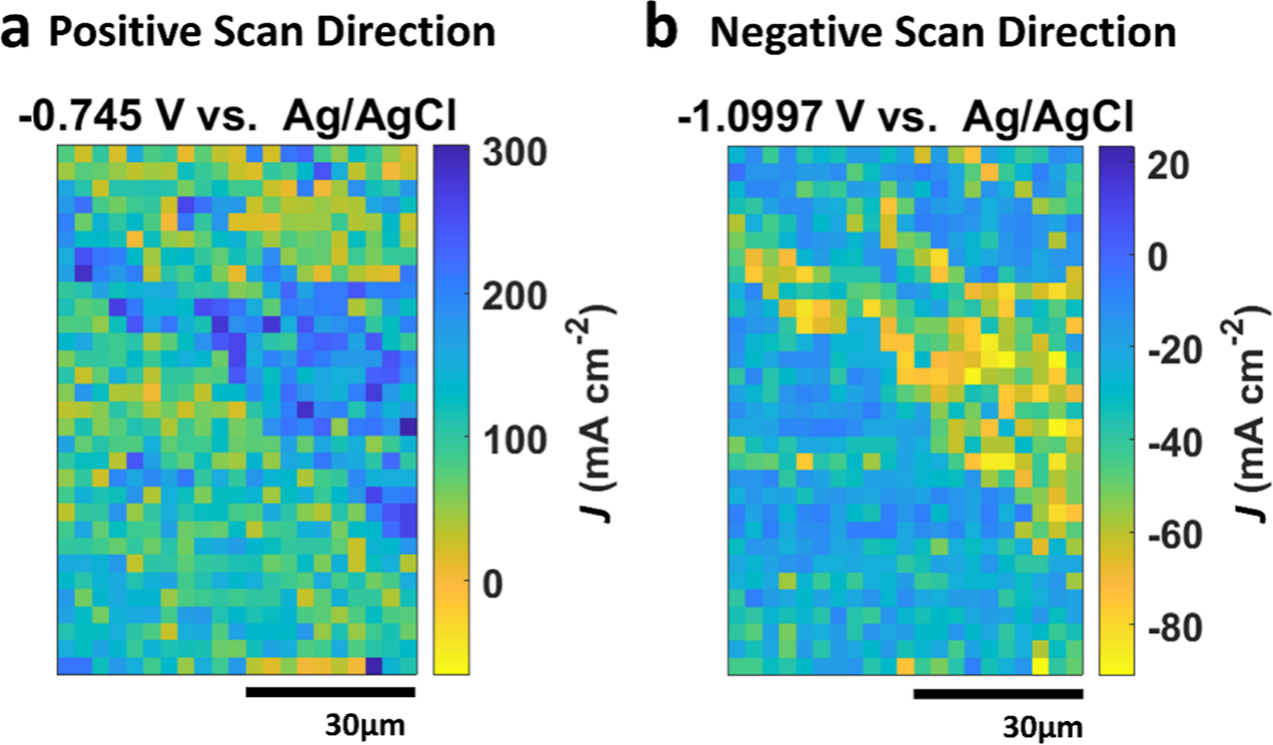
Spatially resolved current density (*J*) maps of a 60 × 90 μm^2^ SECCM scan over the Zn–Al alloy: (a) −0.745 V vs Ag/AgCl equipotential frame in the positive scan direction and (b) −1.099 V vs Ag/AgCl equipotential frame in the negative scan direction.

The collection of the 651 CVs from the SECCM measurements (Figure 2a) were replotted as 701 spatially resolved equipotential electrochemical maps, which were further transformed into an electrochemical movie (Movie S1 in the Supporting Information).^35,46^ At most *E*_app_ values, the electrochemical maps displayed a relatively homogeneous current density (see Figure S5 in the Supporting Information) across the various microstructures on the Zn–Al alloy surface (see Figure 2). However, it is interesting to note that there was a narrow range of *E*_app_ in each scan direction that showed distinctive heterogeneity in the electrochemical map related to the microstructure of the alloy surface. This range extended from −0.88 to −0.70 V in the positive direction (i.e., during the initial stages of metal dissolution) and from −1.02 to −1.20 V in the negative direction (i.e., during metal redeposition and the ORR). Figure 3a,b shows representative electrochemical maps taken from within these ranges, which were obtained at −0.745 V in the positive scan direction and −1.1 V in the negative scan direction, respectively.

Figure 3a depicts anodically more reactive areas, represented by blue-colored pixels in the range of 200–300 mA cm^–2^, in comparison to the surrounding regions. Similarly, Figure 3b illustrates cathodically more reactive areas, denoted by yellow pixels in the range of −60 to −80 mA cm^–2^. A preliminary comparison with Figure 2 suggests that these areas roughly correspond to the area containing the aforementioned dark nanospots (i.e., the Al-rich α phase). To gain a more comprehensive understanding of the structure–activity relationship of the Zn–Al alloy at the nanoscale, we conducted further in-depth SECCM data analysis and quantitative elemental composition assessment below.

**Figure 4:**
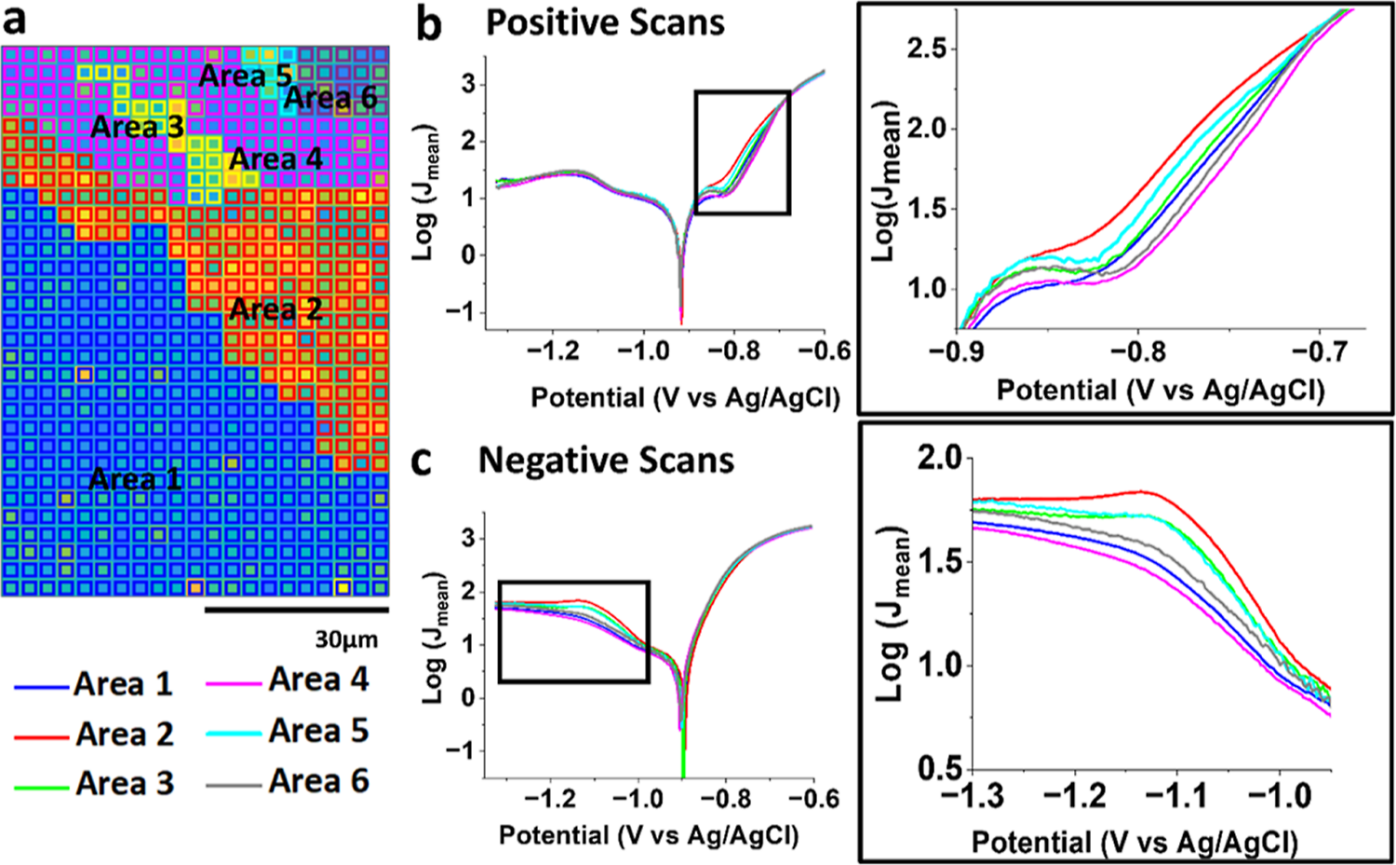
(a) Equipotential map of *J* at −1.099 V vs Ag/AgCl divided into six areas (from Area1 to Area6) based on the observed microstructural features under the SEM image in Figure 2. Averaging individual measurements from each area yields (b) Tafel plots from −1.33 to −0.6 V vs Ag/AgCl in the positive direction and (c) Tafel plots from −1.33 to −0.6 V vs Ag/AgCl in the negative direction at a scan rate of 0.5 V s^–1^.

Based on the observation of the SEM image in Figure 2, where the imaged area overlaps with the SECCM scan (Figure 3), the electrochemical map was divided into six distinct regions, Area1–Area6, as presented in Figure 4a. The number of pixels for each area is as follows: Area1 (*N*_A1_ = 331), Area2 (*N*_A2_ = 165), Area3 (*N*_A3_ = 23), Area4 (*N*_A4_ = 103), Area5 (*N*_A5_ = 9), and Area6 (*N*_A6_ = 20) accounting for a total of 651 pixels. The group of CVs in each Area1–6 was averaged and presented as semilogarithmic current density versus potential (Log|*J*|–*E*) plots, in the positive and negative scan directions, as shown in Figure 4b,c, respectively. The averaged CVs in individual areas are also available in Figure S6 in the Supporting Information. Additionally, Figure S7 in the Supporting Information showcases the averaged CVs for each area along with their respective standard deviation curves.

As predicted from the SECCM map, all six areas mostly exhibited identical Log|*J*|–E plots in the positive scan direction (Figure 3b), except within the potential range of −0.88 to −0.7 V, attributable to the formation of metal (oxy)hydroxide complexes prior to the onset of metal dissolution.^47^ At the selected potential of −0.83 V, Log|*J*| increased in the following order: Area4 ≈ Area6 ≈ Area1 < Area3 ≈ Area5 < Area2. Specifically, the mean *J* value of Area2 at −0.83 V was 94% higher than that of Area4, which displayed the lowest reactivity at that particular potential. Additionally, we conducted further analysis on these plots to assess corrosion parameters in individual areas, such as corrosion potential (*E*_corr_) and corrosion current (*I*_corr_), which are often used to indicate the corrosion activity of the material (see Table S1 in the Supporting Information). The analysis revealed no significant variations in any of the corrosion parameters.

In the negative scan (Figure 3c), the Log|*J*|–*E* plots displayed a variation in Log|*J*| within the voltage range of −1.3 to −0.95 V, while maintaining consistency across all areas for the rest of the plot. At the selected potential of −1.16 V, Log|*J*| increased in the same order as the results from the positive scan: Area4 ≈ Area6 ≈ Area1 < Area3 ≈ Area5 < Area2. Specifically, at the selected potential of −1.16 V, Area2 exhibited the highest (or most negative) *J*, showing 95% variation compared to Area4, which demonstrated the lowest *J*. Notably, as seen more clearly in Figure S6 in the Supporting Information, there is an additional reduction process in Area2, Area3, and Area5 at −1.13 V in the negative direction. Considering the matching order of Log|*J*| magnitude and the difference in *J* between Area2 and Area4 at both −0.83 V in the positive direction and −1.16 V in the negative direction, it suggests that there is a correlation between the initial metal dissolution process and subsequent metal redeposition (along with the ORR). These observations are further considered in the sections below through quantitative elemental analysis.

### Identical-Location Energy Dispersive X-ray Spectroscopy Analysis

**Figure 5:**
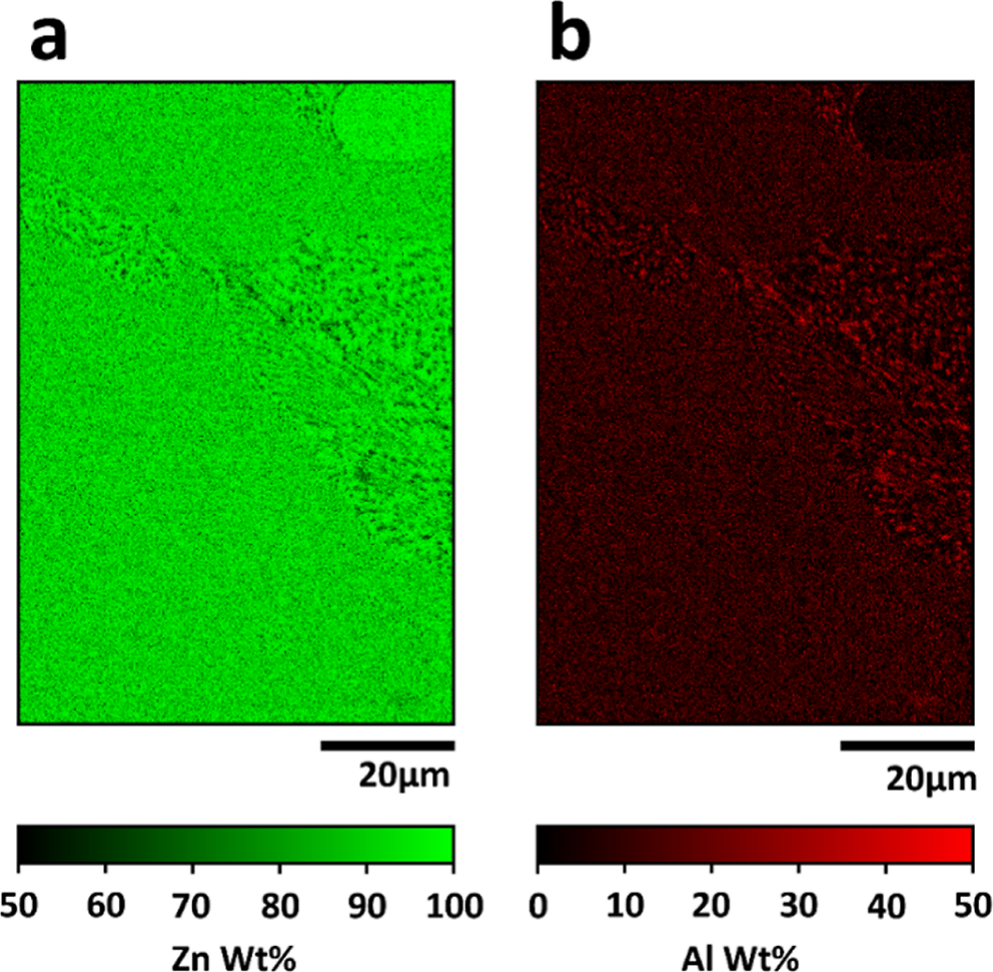
EDS layered image of the SECCM scan area illustrating the compositional distribution along microstructural variations of (a) Zn and (b) Al, presented as weight percentages (wt. %).

To quantitatively assess the distribution of Zn and Al across the distinctive surface microstructure, EDS maps of the entire SECCM scan area were initially acquired, as shown in Figure 5, providing the compositional distribution in weight percent (wt. %) of Zn (Figure 5a) and Al (Figure 5b). These maps cover the same area of the Zn–Al alloy surface depicted in the SEM images presented in Figure 2, as well as the SECCM maps in Figures 3 and 4.

**Figure 6:**
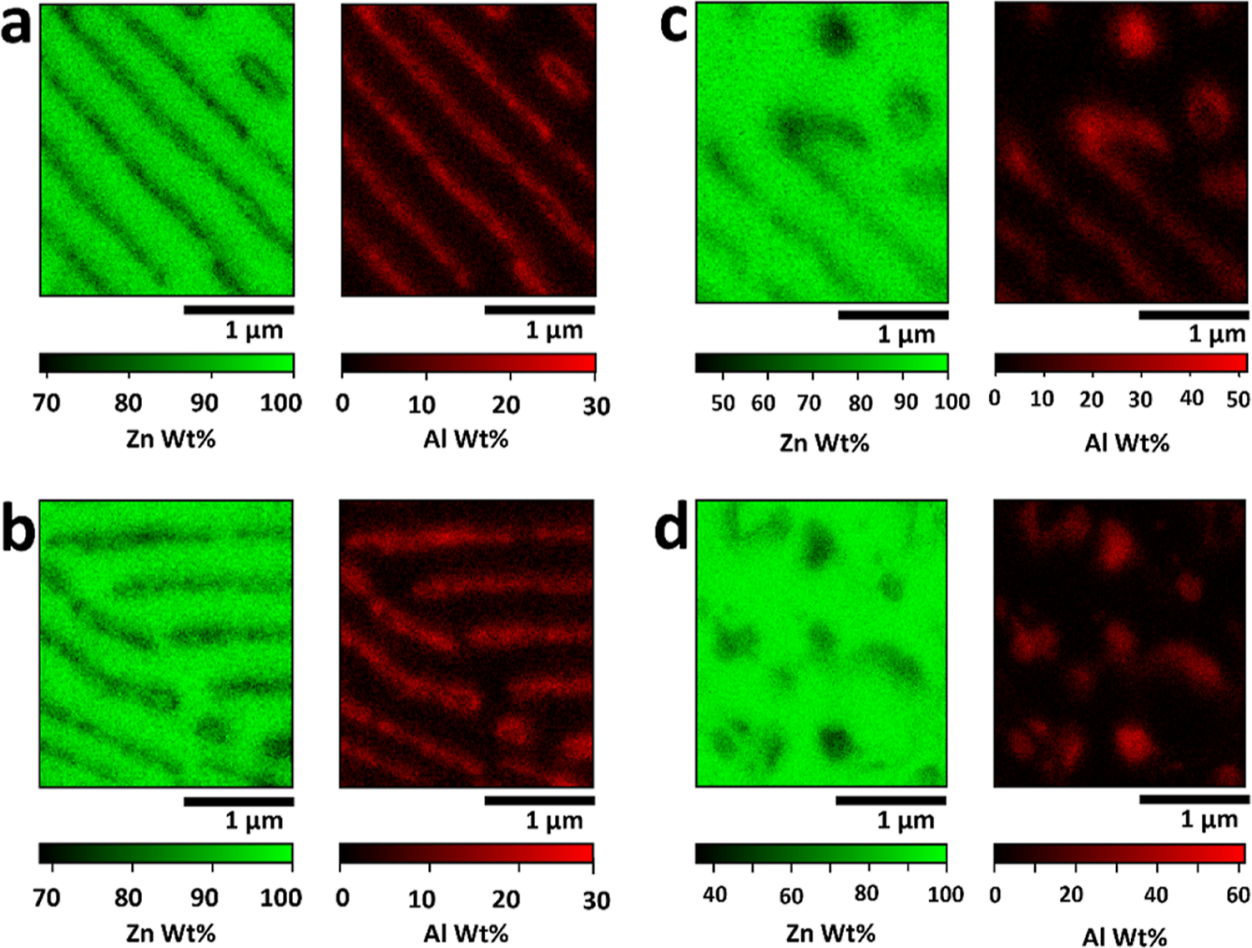
EDS maps of the SECCM scan area with the quantitative composition of the identified microstructures: (a) lamellar structure of Area1 and Area4, (b) GB at Area3 consisting of lamellar structures, (c) interface between the lamellar structure of Area1 and Al-rich nanospots of Area2, and (d) Al-rich nanospots of Area2.

Comparing identical-location SEM and EDS maps in Figures 2 and 5, respectively, with the areal analysis of SECCM maps in Figure 4, Area1 and Area4 exhibit relatively homogeneous Zn and Al compositional distribution at the scale of measurement in EDS in Figure 5, which corresponds to the lamellar structure with the layers of Zn-rich β phase and ZnAl eutectic γ phase (Figure 2b,d). Notably, it becomes evident that Area2 and Area5, which exhibited higher electrochemical activities (e.g., higher currents) compared to other areas (Figure 4b,c), directly overlap with regions containing nanospots with a high Al composition (Figure 2d,e), ranging from 35 to 50 wt. % (Figure 5b), corresponding to Al-rich α phase. Area6 contains the highest Zn composition in the alloy, i.e., dendritic Zn-rich β phase (Figure 2a,c). Consequently, a high-resolution EDS analysis was further conducted for individual microstructures of the Zn–Al alloy surface (Figure 6).

The distribution of Zn and Al in four distinct areas (Figure 6), representing the lamellar structure (Figure 6a), grain boundaries (Figure 6b), and Al-rich spots (Figure 6c,d), was further investigated with high-resolution EDS mapping. The obtained point-EDS spectra within each map were analyzed for Zn and Al composition and phase identification in the local region (see Section S8 in the Supporting Information) and summarized in Table 1, corresponding to Area1 to Area6, as defined in Figure 4.

**Table 1:** Elemental Compositions of Microstructures from EDS Analysis as Weight Percentages (wt.%)

		elemental composition (wt. %)	
microstructure	phase	Zn	Al
Lamellar structure	β	97.4	2.6
(Area1 & Area4)	γ	84.7	15.3
GB	β	97.4	2.6
(Area3)	γ	85.3	14.7
nanospots	β	97.6	2.4
(Area2 and Area5)	α	67.9	32.1
dendritic island	β	98.5	1.4
(Area6)			

Figure 6a represents the lamellar structures corresponding to Area1 and Area4, which have been identified as the β and γ phases. The elemental composition aligns with the theoretical predictions based on the Zn–Al alloy phase diagram,^5,42^ in which the β phase corresponds to an elemental composition of 99% of Zn and 1% of Al (based on weight percentage). Further, the γ phase consists of 73–78% Zn and 23–26% Al, on a weight percentages basis. Figure 6b illustrates a GB in Area3, where two grains of the lamellar structure in Area4 meet (as shown in Figure 2b). As seen in Figure 6b and Table 1, Area3 also consists of the β and γ phases with composition profiles almost identical to those in Area1 and Area4. This validates that the elevated activities observed in Area3 compared to Area1 and Area4 (Figure 4b,c) are due to the presence of a GB, which likely has a high density of defects nearby, which impacts the electrochemical properties.^48^Figure 6c visualizes the interface between Area2 and Area5, clearly showing that the Al-concentrated region in Area2 is the ZnAl eutectic β phase (2.4 wt. % Al), while the nanospots in Area5 represent the Al-rich α phase (32 wt. % Al). The remaining regions were confirmed to have the presence of the β phase (below 2.4 wt. % Al). Figure 6d further displays 13 Al-rich α phase nanospots in Area2, with Al contents ranging from 33 to 55 wt. %, evidencing that the high electrochemical activities in both Area2 and Area5 are attributable to the Al-rich α phase nanospots. The dendritic Zn-rich island (see Figure S11 and Table S5 in the Supporting Information) identified as the β phase contains Al below 5% across the island.

Under the ORR (i.e., 2H_2_O + O_2_ + 4e^–^ → 4OH^–^), the local pH increases in unbuffered neutral media, adding complexity to the metal dissolution in Zn–Al alloy.^38,49^ The rate of Al dissolution in alkaline media is higher than that of Zn at an equivalent pH.^4,5^ In high pH conditions, zincite ZnO and/or Zn(OH)_2_ are thermodynamically favored on Zn, ultimately becoming soluble as Zn(OH)_3_^–^ and Zn(OH)_4_^2–^ with increasing pH. For Al in an alkaline solution, the native Al_2_O_3_ and Al(OH)_3_ films are less stable than their Zn counterparts, and the dominant species become AlO_2_^–^, which is highly soluble. Due to the lower stability of the Al-rich phase in an alkaline environment induced by the ORR in unbuffered neutral media,^5,50^ particularly in Area2 and Area5, which contain Al-rich α phase nanospots, variations in anodic and cathodic processes were observed at −0.83 V in the positive direction and at −1.1 V in the negative direction, respectively (Figure 4c). The additional process at −1.1 V in the negative direction resulted from the redeposition of dissolved aluminum products during the electrochemical reaction, which coincides with the ORR.^3^

### Variation of Electrochemical Activity within Area2

**Figure 7:**
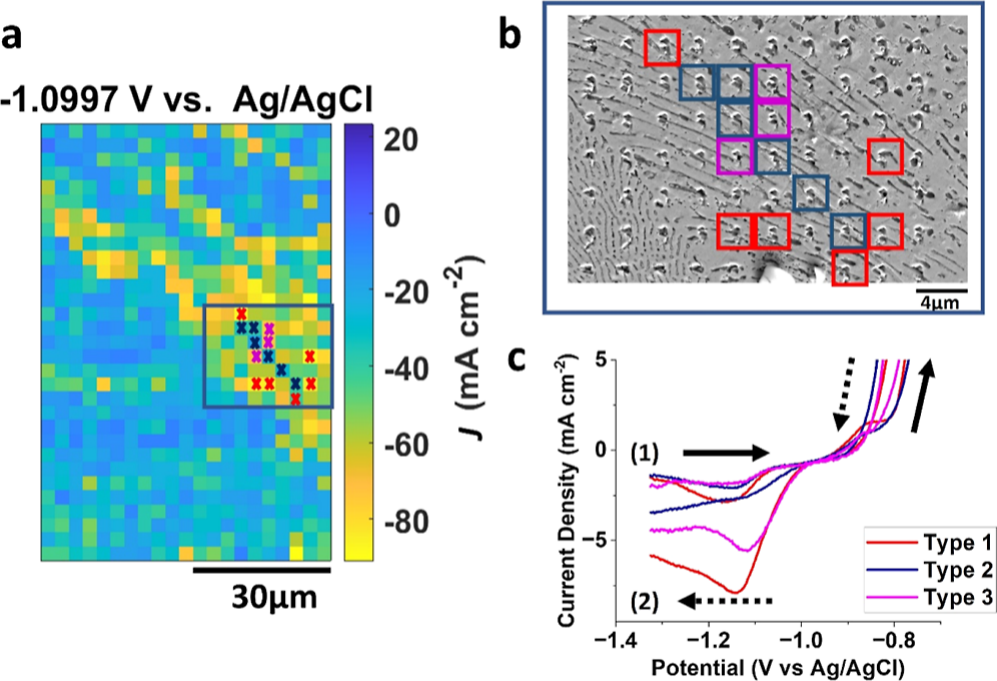
(a) Equipotential map of *J* at −1.1 V vs Ag/AgCl in Figure 2b, representing measurements within the region of Area2. (b) SEM image of the area indicated by the blue rectangle within Area2 in (a). Note that type 1 is marked with red square, type 2 is marked with blue square, and type 3 is marked with magenta square. (c) Averaged CVs corresponding to the varied activities marked in both (a,b), with (1) representing initial positive sweep direction and (2) representing the negative sweep direction.

As the size of the SECCM probe determines the spatial resolution in the hopping scan mode,^51^ structure–activity relationships can be more clearly revealed when the size of the microstructure is comparable to or larger than the probe size. In Area2, where the nanospots are sparsely placed, we observed relatively high heterogeneity in electrochemical activity at −1.1 V in the negative scan direction. When examining electrochemical heterogeneity within Area2 with the corresponding SEM image, it was observed that a microstructure, depicting a scratch, shows relatively low *J* at −1.1 V (*J*_–1.1 V_) within Area2, i.e., lower than [the averaged *J* at −1.1 V (*J*_ave_) – 3 × S.D.] (Type 2). Consequently, 9 additional points adjacent to the scratch were chosen for individual analysis, either those with *J*_–1.1 V_ near *J*_ave_ (Type 3) or those with a *J*_–1.1 V_ exceeding 3 standard deviations (Type 1). In brief, the selected 15 individual measurements in Area2 (Figure 7a,b) were categorized into Types 1, 2, and 3, depending on their variation from the averaged *J* at −1.1 V (*J*_ave_) in Area2: type 1 (*N* = 6) represents cases where the *J* at −1.1 V (*J*_–1.1 V_) is higher than (*J*_ave_ + 3 × S.D.); type 2 (*N* = 6) corresponds to cases where the *J*_–1.1 V_ is lower than (*J*_ave_ – 3 × S.D.); type 3 (*N* = 3) includes cases where *J*_–1.1 V_ is close to *J*_ave_. The averaged CVs for each type are presented in Figure 7c. The individual CVs corresponding to the selected 15 points that categorized into the 3 types are shown in Figure S12 in the Supporting Information.

We also observed the additional reduction process in the voltammetric profiles observed from −1.0 to −1.33 V in the negative direction, which aligns with our observations from the total area of the SECCM scan (Figure 4, vide supra). Both type 1 and type 3 showed a peak near 1.13 V, attributable to the redeposition of soluble Al product alongside the ORR and also is in good agreement with the average response from Area2, with type 1 exhibiting a 45% higher peak current than type 3. Notably, SEM images of the corresponding areas revealed that the meniscus from the SECCM probe directly covered a larger area of the Al-rich α phase in type 1 measurements than in type 3. In type 2, an interesting observation was made: the peak at −1.1 V did not appear. In type 2, there was an additional microstructure, a minor scratch on the alloy surface produced during sample preparation, which increased the roughness of the surface as well as the physical defect density. As the scratch alters both the surface morphology and structure, it may have a more significant impact on the metal dissolution/redeposition process that is surface- and mass transport-sensitive.

## Conclusions

In summary, this study has unveiled intricate details of the electrochemical behavior of the as-cast Zn-4 wt. %Al alloy. Through the synergistic application of SECCM, SEM, and EDS techniques, significant observations were made. The SECCM scans highlighted anodic and cathodic regions of elevated reactivity within the alloy. These localized reactivity variations correspond, to some extent, with the regions housing dark nanospots, representing the Al-rich α phase, as evident from subsequent analysis.

Further segmenting the electrochemical map into six distinct areas based on SEM-imaged microstructural features allowed for an in-depth examination of electrochemical properties in these zones. While mostly analogous, comparisons of averaged voltammograms and semilogarithmic log|*J*|–*E* plots in the individual areas have illustrated some complexity of electrochemical reactions taking place across the surface of the alloy. In the negative sweep direction, particularly at −1.16 V, the sequence of reactivity mirrored that observed in the positive sweep direction, with one region exhibiting the highest reactivity (Area2) and another the lowest (Area4). Moreover, we noted an additional reduction process at −1.13 V in the negative scan, particularly in areas containing nanospots and a GB. The observed heterogeneity in electrochemical behavior within the alloy is possibly associated with the initial metal dissolution process and subsequent redeposition, in conjunction with the ORR, which locally induces an alkaline environment under unbuffered conditions. With a detailed elemental analysis conducted through EDS, it was identified that the variations in electrochemical behavior among the areas are attributable to the Al-rich α-phase, which contains Al from 30 to 50%, inducing local Al dissolution during the ORR.

To elucidate the nuanced electrochemical variations within a specific area, we further conducted a detailed assessment within Area2. We categorized 16 individual measurements in Area2 into three types. With identical-location SEM analysis at high magnification, it was further confirmed that the presence of an additional reduction process in voltammetric profiles in the negative scan direction is attributable to the Al-rich α phase in the nanospots, reinforcing the potential linkage between the initial metal dissolution process and redeposition, in the presence of the ORR.

In conclusion, these findings emphasize the critical role of microstructural features in governing the electrochemical reactivity of the Zn–Al alloy. This work opens avenues for further investigations in the realm of alloy behavior and provides insights for engineering materials with tailored electrochemical properties. The interplay between microstructure and electrochemical performance is a compelling area for future research, offering prospects for the development of advanced materials for various applications.

## Supporting Information Available

SEM images of the probe and the as-cast Zn–Al alloy; SEM images of the scanned alloy surface in BSE mode; additional equipotential SECCM maps; averaged CVs for Area1 to Area6; results from Tafel analysis for Area1 to Area6; and quantitative elemental analysis of Area1 to Area6 via EDS (PDF)
Spatially-resolved electrochemical (current−voltage) movie (MP4)
