# Supplementary material for: High Throughput Correlative Electrochemistry-Microscopy Analysis on a Zn–Al Alloy
Source: ACS Phys Chem Au. 2024 May 10;4(4):375–84. doi: 10.1021/acsphyschemau.4c00016 (PMC11274284; doi:10.1021/acsphyschemau.4c00016)
Supplement: Supplementary file 1 — pg4c00016_si_001.pdf [file pg4c00016_si_001.pdf]

High Throughput Correlative Electrochemistry-Microscopy Analysis on a Zn-Al alloy

Gunani Jayamaha,[1] Levi Tegg,[2] Cameron L. Bentley,[3] Minkyung Kang[1*]

[1]School of Chemistry, The University of Sydney, Camperdown 2006 NSW, Australia

[2]School of Aerospace, Mechanical and Mechatronic Engineering, The University of Sydney, Camperdown 2006 NSW, Australia

[3]School of Chemistry, Monash University, Clayton 3800 VIC, Australia

E-mail: minkyung.kang@sydney.edu.au (M.K.)

Table of Contents

Section S1. Probe and substrate preparation S2
Section S2. SEM image of as-cast Zn-Al alloy S3
Section S3. SEM images in backscattered electron (BSE) mode S4
Section S4. Movie captions S5
Section S5. Additional equipotential frames for SECCM scan S6
Section S6. Complete cyclic voltammograms for Area1 - Area6 .... S7
Section S7. Tafel analysis S8
Section S8. Quantitative elemental analysis of Area 1 to 6 .... S9
Section S9. Individual CVs for Type 1, Type 2 and Type 3 categories within Area2 .... S13
References S14

Section S1. Probe and substrate preparation

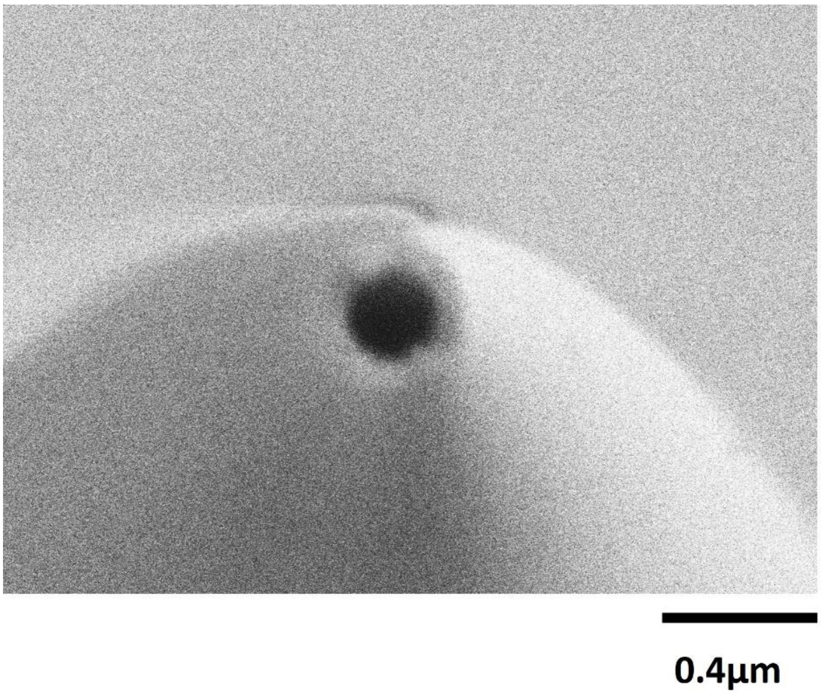

Figure S1 SEM image of a representative pipette tip with an outer diameter of approximately 400 nm, used for the SECCM scans.

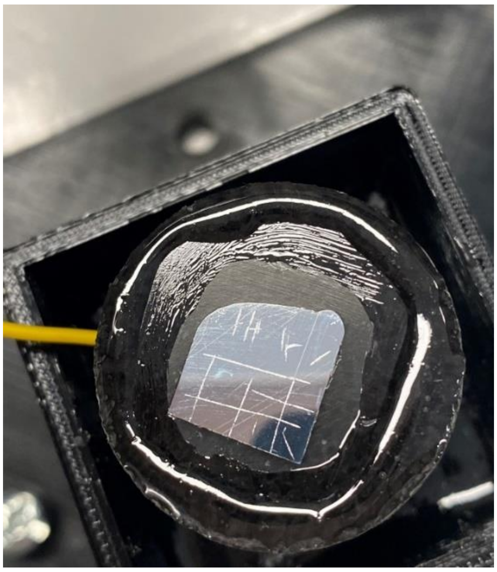

Figure S2 Zn-Al alloy sample mounted on a carbon-mixed resin with a resin ring to hold the oil layer for SECCM scanning.

Section S2. SEM image of as-cast Zn-Al alloy

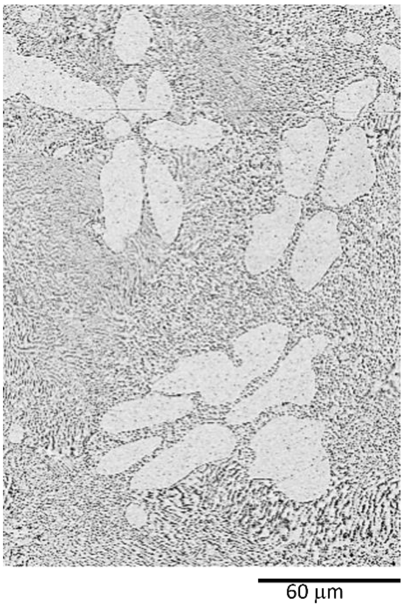

Figure S3 SEM image of the hypo-eutectic Zn-4wt.%Al alloy microstructure, showing dendritic Zn-rich islands surrounded by Zn and Al lamellar structures.

For hypo-eutectic alloys at around 416°C, a mixture of β(Zn) phase and liquid (L) is created. For Zn-4.wt%Al, this mixture exists until the temperature drops to 382°C, where a eutectic transformation occurs, resulting in the formation of β(Zn) + γ(ZnAl). Below 382°C, the alloy consists of a mixture of β(Zn) phase and eutectic (β(Zn) + γ(ZnAl)) until the temperature drops to 275°C. At this temperature, the eutectoid transformation occurs, forming γ(ZnAl)), α(Al) + β(Zn), and the alloy now consists of a mixture of β(primary, rich in Zn) and eutectoid (α + β). At temperatures below 275°C, the alloy consists of a mixture of β(primary, rich in Zn) and eutectoid (α + β). [1-3]

Section S3. SEM images in backscattered electron (BSE) mode

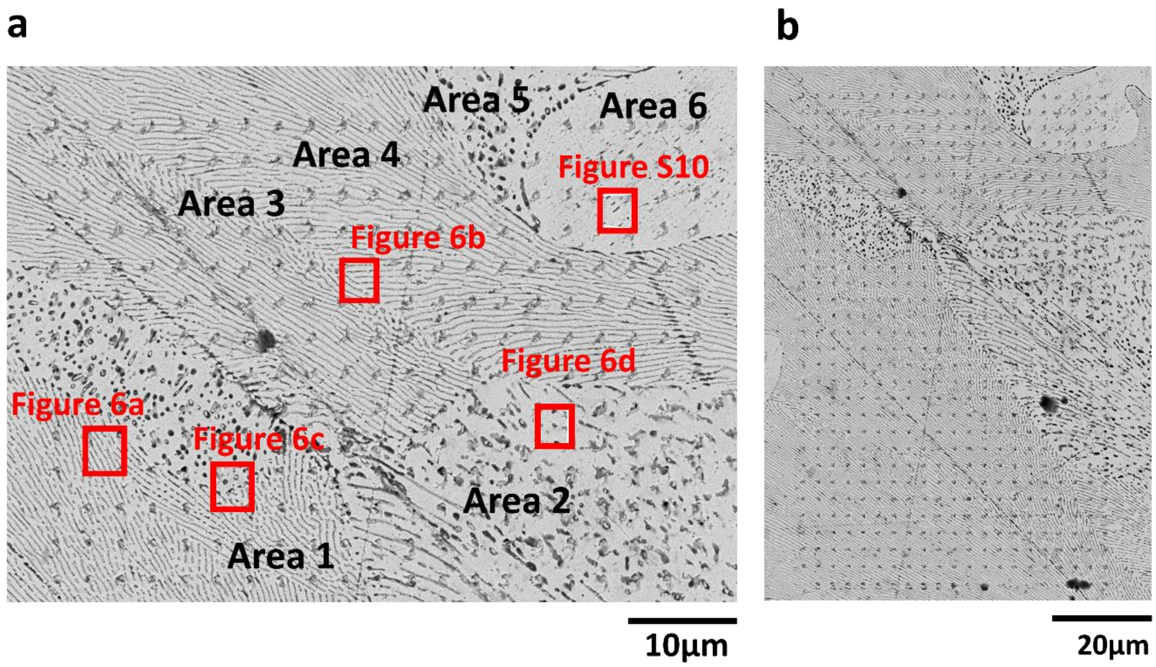

Figure S4 (a) SEM backscattered electron (BSE) mode image of the selected section of the SECCM scan area, defining areas from Area1 to Area6. The contrasting shades illustrate compositional variations. Red squares denote the locations for quantitative compositional analysis in Figure 6 in the main text and Figure S10 in the Supporting Information. (b) SEM BSE mode image of a complete SECCM scan area highlighting pronounced compositional variations among various microstructures of the Zn-Al alloy.

Section S4. Movie captions

Movie S1. Spatially-resolved electrochemical (current−voltage) movie (21 × 31 pixels over a 60 × 90μm^2 area, hopping distance 3 μm) obtained with the voltammetric (υ = 0.5 V s^{-1}) hopping mode SECCM configuration, visualizing electrochemical activity of the as-cast Zn-Al alloy surfaces. The micropipette probe (d_{tip} = 400 nm) was equipped with Ag/AgCl QRCEs and filled with 10 mM NaCl. Data extracted from Movie S1 were used to construct Figure 3 in the main text as well as Section S5 in the Supporting Information. The data presented are not interpolated.

Section S5. Additional equipotential frames for SECCM scan

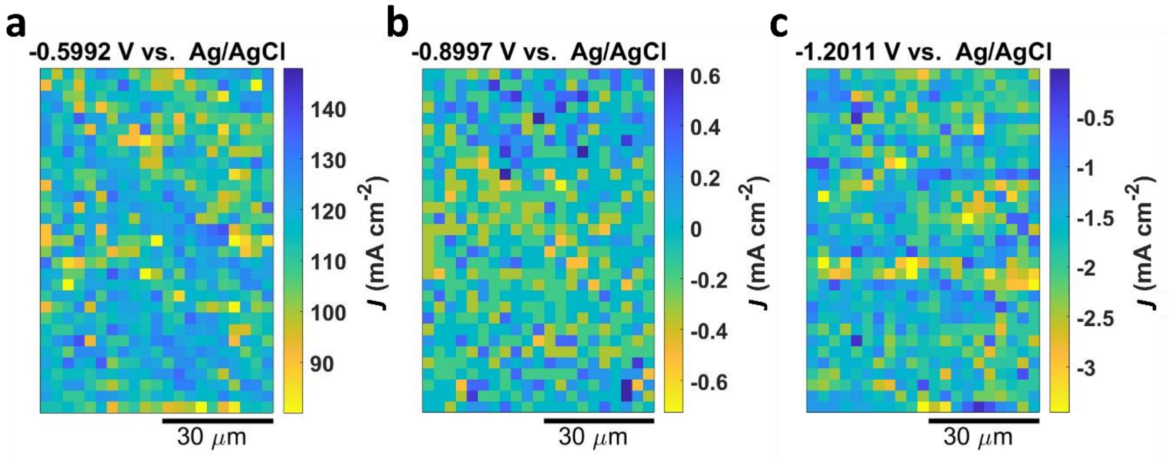

Figure S5: Equipotential frames obtained from the SECCM movie, (a) -0.60 V vs Ag/AgCl in the positive direction of the scan, (b) -0.90 V vs Ag/AgCl in the positive direction and (c) -1.20 V vs Ag/AgCl in the negative direction which do not represent any microstructure- based activity variations.

Section S6. Complete cyclic voltammograms for Area1 - Area6

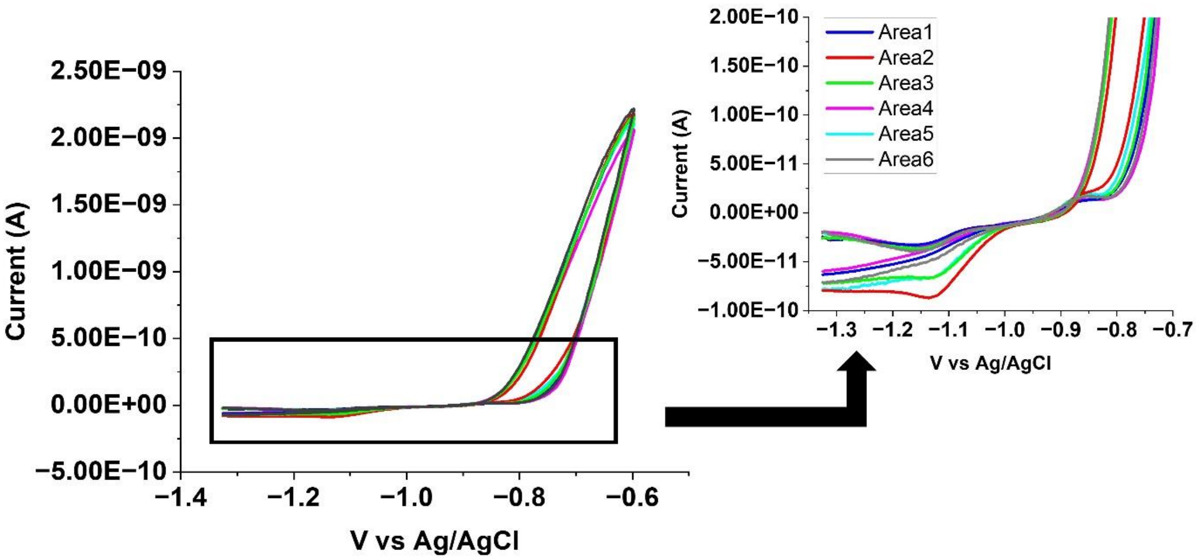

Figure S6 Averaged cyclic voltammograms (CVs) for Area1 to Area6.

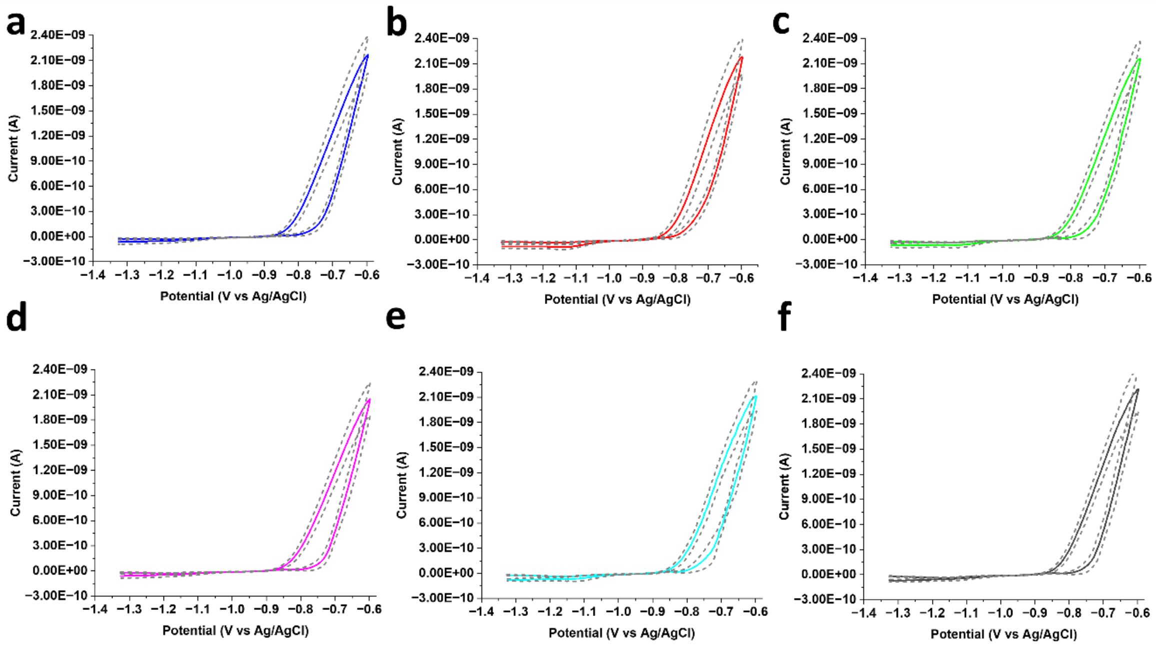

Figure S7 Averaged cyclic voltammograms for (a) Area1 (b) Area2 (c) Area3 (d) Area4 (e) Area5 and (f) Area6 (colored solid lines) with standard deviation (grey dotted lines).

Section S7. Tafel analysis

Table S1 The corrosion potential (E_{corr}) or corrosion current density (I_{corr}) from Area1 to Area6 obtained by analyzing Tafel plots in Figure 4 in the main text.

**Area based on microstructural variation**	**E_{corr} (V)**	**I_{corr} (mAcm^{-2})**
Area1	-0.916	0.097
Area2	-0.916	0.106
Area3	-0.919	0.089
Area4	-0.916	0.090
Area5	-0.918	0.115
Area6	-0.920	0.093

Section S8. Quantitative elemental analysis of Area 1 to 6

Elemental composition of Lamellar structures – Area1 & Area4

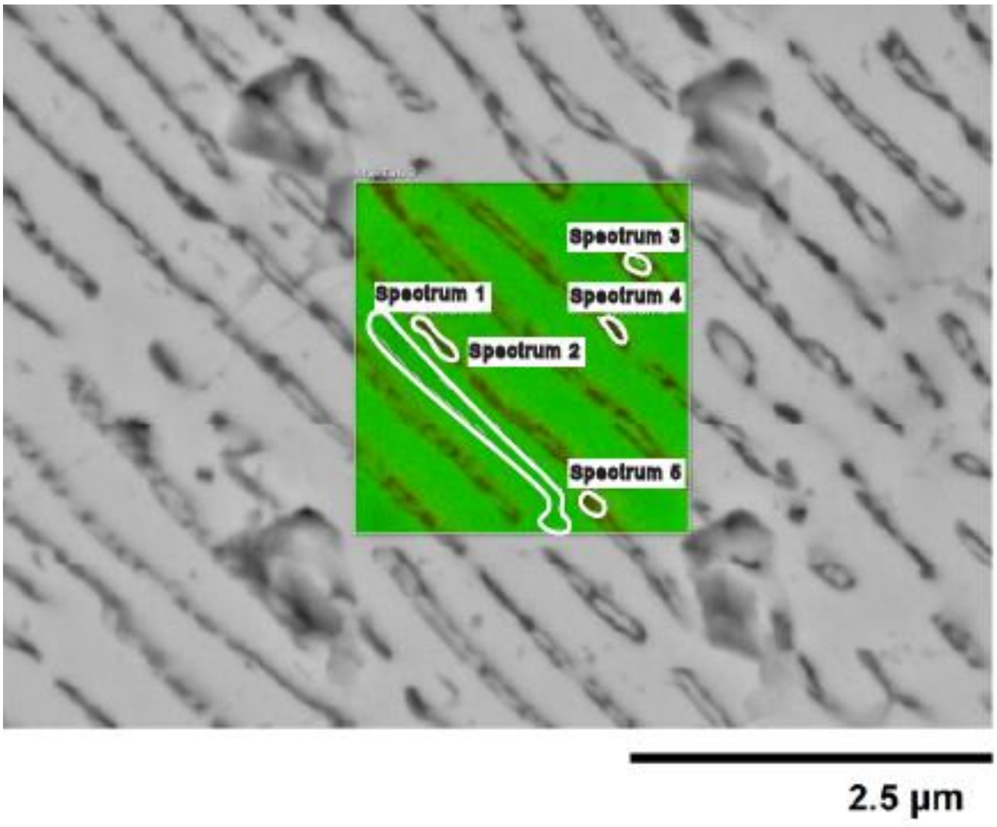

Figure S8 EDS analysis conducted on lamellar structures representing Area1 & Area4, the quantitative elemental composition of Spectrum1 – Spectrum5 are tabulated in Table S2

Table S*2* The averaged elemental composition in weight percentages (wt.%) of the lamellar structures representing Area1 & Area4 and the quantitative elemental composition of Spectrum1 – Spectrum5 shown in Figure S7

Microstructural Features	Spectrum	Elemental Composition (wt.%)		Averaged Elemental Composition (%)	
		Zn	Al	Zn	Al
Lighter grey phase	1	97.4	2.6		
Dark grey phase	2	85.0	15.0	84.7	15.3
	3	88.7	11.3		
	4	82.1	17.9		
	5	82.9	17.1		

Elemental composition of the grain boundary – Area3

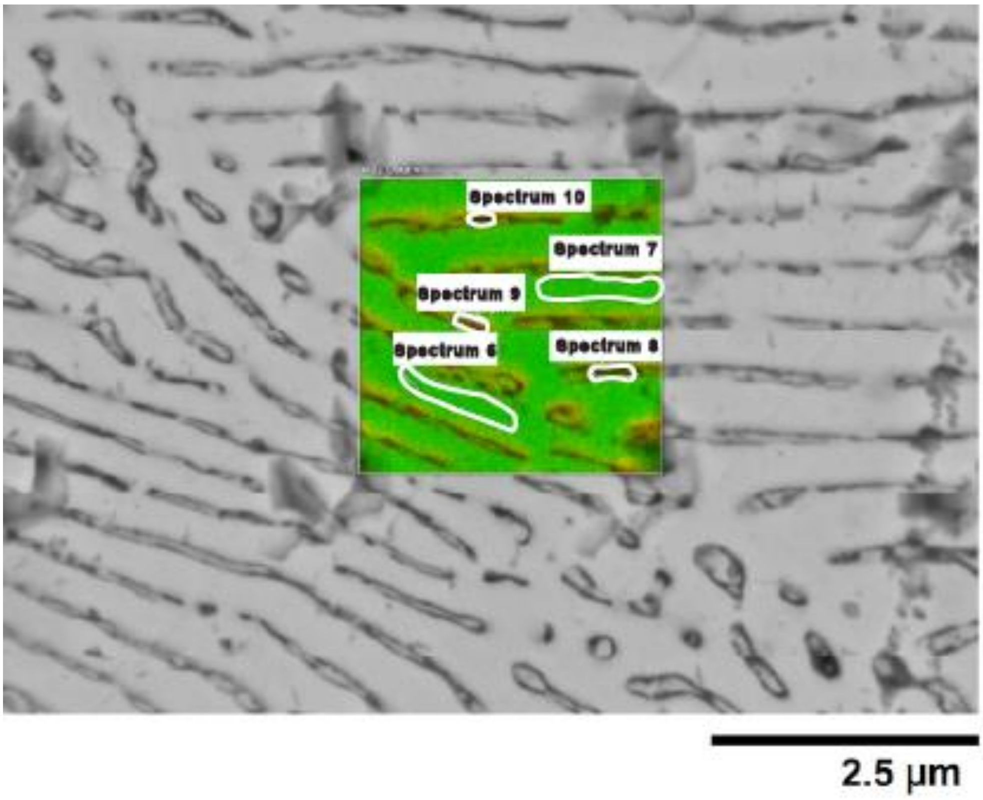

Figure S9 EDS analysis conducted on the grain boundary representing Area3, the quantitative elemental composition of Spectrum6 – Spectrum10 are tabulated in Table S3

Table S*3* The averaged elemental composition in weight percentages (wt.%) of the grain boundary representing Area3 and the quantitative elemental composition of Spectrum6 – Spectrum10 shown in Figure S8

Microstructural Features	Spectrum	Elemental Composition (wt.%)		Averaged Elemental Composition (%)	
		Zn	Al	Zn	Al
Lighter grey phase	6	97.2	2.8	97.4	2.6
	7	97.7	2.3		
Dark grey phase	8	87.0	12.9	85.3	14.7
	9	86.5	13.5		
	10	82.3	17.7		

Elemental composition of Al rich area – Area2 & Area5

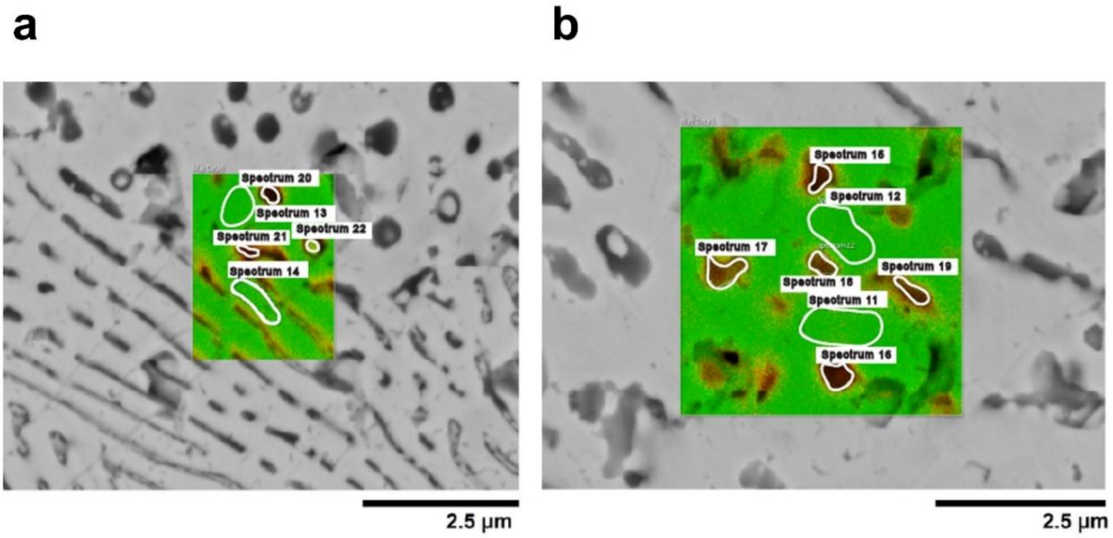

Figure S10 EDS analysis conducted on Al rich microstructural area representing Area2 & Area5, locations specific to (a) Spectrum13,14,20-22 and (b) Spectrum 11,12, 15-19 the quantitative elemental composition of each spectrum are tabulated in Table S4

Table S*4* The averaged elemental composition in weight percentages (wt.%) of the grain boundary representing Area3 and the quantitative elemental composition of Spectrum6 – Spectrum10 shown in Figure S8

**Microstructural Features**	**Spectrum**	**Elemental Composition (wt.%)**		**Averaged Elemental Composition (%)**	
		**Zn**	**Al**	**Zn**	**Al**
	11	97.9	2.1		
	12	98.9	1.1		
Lighter grey phase	13	98.5	1.5	98.0	2.0
	14	96.7	3.3		
	15	66.7	33.3		
	16	64.3	35.7		
	17	76.7	23.3		
	18	77.5	22.5		
Dark grey phase	19	75.1	24.9	70.9	29.1
	20	64.9	35.1		
	21	70.9	29.1		

Elemental composition of Zn rich island – Area6

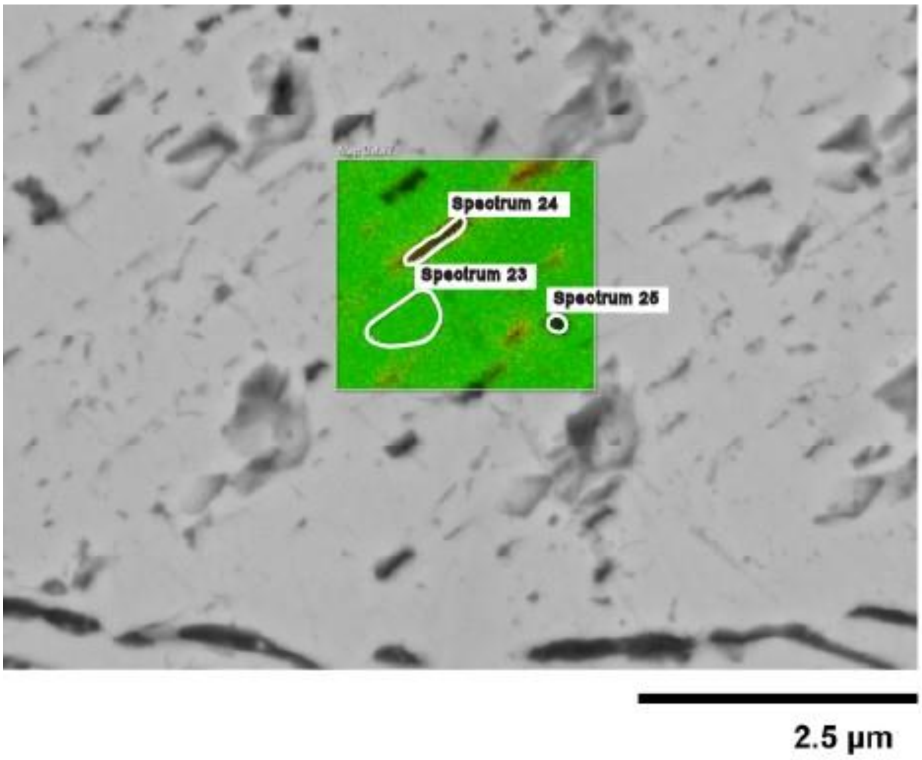

Figure S11 EDS analysis conducted on lamellar structures representing Area1 & Area4, the quantitative elemental composition of Spectrum1 – Spectrum5 are tabulated in Table S2

Table S5 The averaged elemental composition in weight percentages (wt.%) of the grain boundary representing Area3 and the quantitative elemental composition of Spectrum6 – Spectrum10 shown in Figure S8

**Microstructural Features**	**Spectrum**	**Elemental Composition (wt.%)**		**Averaged Elemental Composition (%)**	
		**Zn**	**Al**	**Zn**	**Al**
Lighter grey phase	23	98.6	1.4		
Dark grey phase	24	93.1	6.9	94.6	5.4
	25	96.1	3.9		

Section S9. Individual CVs for Type 1, Type 2 and Type 3 categories within Area2

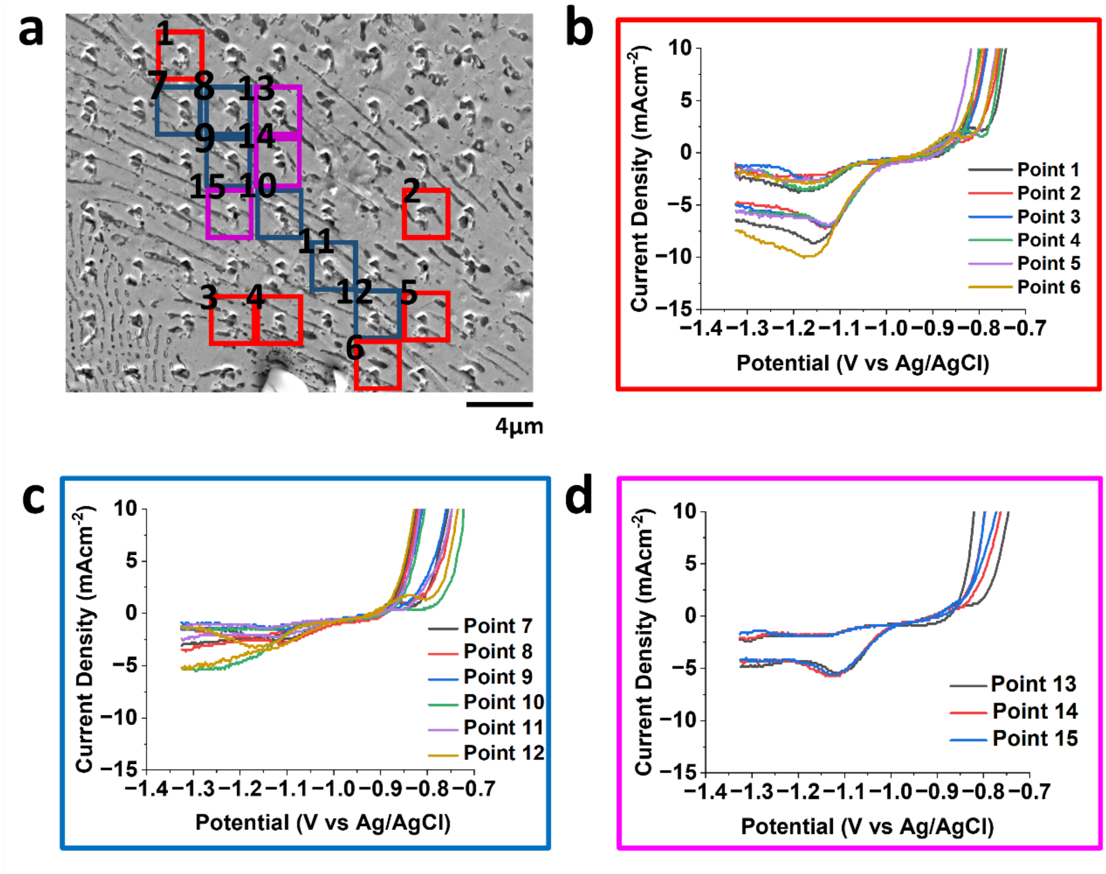

Figure S12 (a) SEM image of the selected SECCM scan of Area2 as in Figure7. Note that Type1 is marked with red square, Type 2 is marked with blue square and Type 3 is marked with magenta square. (b) Individual CVs corresponding to Points 1 - 6 of Type 1 category. (c) Individual CVs corresponding to Points 7 - 12 of Type 2 category. (d) Individual CVs corresponding to Points 13 - 15 of Type 3 category.

References

(1) Prosek, T.; Hagström, J.; Persson, D.; Fuertes, N.; Lindberg, F.; Chocholatý, O.; Taxén, C.; Šerák, J.; Thierry, D. Effect of the microstructure of Zn-Al and Zn-Al-Mg model alloys on corrosion stability. *Corrosion Science* **2016**, *110*, 71-81.

(2) Ares, A. E.; Gassa, L. M. Corrosion susceptibility of Zn–Al alloys with different grains and dendritic microstructures in NaCl solutions. *Corrosion Science* **2012**, *59*, 290-306.

(3) Boyer, H. E.; Gall, T. L. Metals handbook; **1985**, *American Society for Metals*.
